# Density Analysis of Enterovirus D68 Shows Viral Particles Can Associate with Exosomes

**DOI:** 10.1128/spectrum.02452-21

**Published:** 2022-02-16

**Authors:** Michael J. Rudy, Christina Coughlan, Alison M. Hixon, Penny Clarke, Kenneth L. Tyler

**Affiliations:** a Department of Neurology, University of Colorado, Aurora, Colorado, USA; b University of Colorado Alzheimer’s and Cognition Center, Aurora, Colorado, USA; c Linda Crnic Institute for Down Syndrome, Aurora, Colorado, USA; d Medical Scientist Training Program, University of Colorado, Aurora, Colorado, USA; e Department of Immunology and Microbiology, University of Colorado, Aurora, Colorado, USA; f Division of Infectious Disease, Department of Medicine, University of Colorado, Aurora, Colorado, USA; g VA Medical Center, Aurora, Colorado, USA; Wuhan Institute of Virology

**Keywords:** density gradient, enterovirus D68, exosomes, high-speed centrifugation, small extracellular vesicles

## Abstract

Enterovirus D68 (EV-D68) is an emerging pathogen which causes respiratory disease and is associated with an acute flaccid myelitis that predominately affects children. EV-D68 can infect motor neurons, causing cell death and a loss of motor control leading to flaccid paralysis. However, it remains unknown how viral particles gain entry into the central nervous system (CNS). Here, we show that three distinct densities of EV-D68 particle can be isolated from infected muscle and neural cell lines (RD and SH-SY5Y) using high-speed density centrifugation to separate cell supernatant. The lowest-density peak is composed of viral particles, which have adhered to the exterior surface of a small extracellular vesicle called an exosome. Analysis of prototypic (historic) and contemporary EV-D68 strains suggests that binding to exosomes is a ubiquitous characteristic of EV-D68. We further show that interaction with exosomes increases viral infectivity in a neural cell line. Analysis of the two higher-density peaks, which are not associated with exosomes, revealed that a significant amount of viral titer in the modern (2014) EV-D68 strains is found at 1.20 g/cm^3^, whereas this density has a very low viral titer in the prototypic Fermon strain.

**IMPORTANCE** Despite the strong causal link between enterovirus D68 (EV-D68) and acute flaccid myelitis (AFM), it remains unclear how EV-D68 gains entry into the central nervous system and what receptors enable it to infect motor neurons. We show that EV-D68 particles can adhere to exosomes, placing EV-D68 among a handful of other picornaviruses which are known to interact with extracellular vesicles. Uptake and infection of permissive cells by virally contaminated exosomes would have major implications in the search for the EV-D68 receptor, as well as providing a possible route for viral entry into motor neurons. This work identifies a novel cellular entry route for EV-D68 and may facilitate the identification of genetic risk factors for development of AFM.

## INTRODUCTION

Enterovirus D68 (EV-D68) is a nonenveloped picornavirus which was first isolated from four children with pneumonia in 1962, and the original strains were named after the corresponding patients (Fermon, Rhyne, Franklin, and Robinson). Over the next 40 years, EV-D68 was rarely linked with human disease, with only 699 reported cases prior to 2013. The first major EV-D68 outbreak occurred in the fall of 2014, with 2,287 reported cases worldwide (1). During this outbreak of EV-D68-associated upper respiratory disease, physicians noted a concurrent increase in the incidence of a polio-like paralysis affecting children, referred to as acute flaccid myelitis (AFM). AFM’s causal association with EV-D68 was strengthened by subsequent contemporaneous spikes in AFM and EV-D68-induced respiratory disease during the fall in 2016 and 2018. Some contemporary strains of EV-D68 consistently induce limb paralysis in mice, fulfilling Koch’s postulates for EV-D68-induced paralytic disease and further strengthening the causal link between AFM and EV-D68 (2). Contemporary clades of EV-D68 are thought to have emerged sometime in the late 1990s (clades A and C), the mid 2000s (clade B), or later (clade D). Compared to the prototypic Fermon strain, all contemporary strains (clades A to D) are missing a 21- to 24-bp region in the 5′ UTR, and all possess genomic deletions and substitutions which have the potential to affect virulence (3, 4). However, it remains unclear what factors enabled this virus to begin causing worldwide respiratory outbreaks or why some contemporary strains seem to be more associated with neurovirulence than others (5).

Binding kinetics and the affinity of EV-D68 for various sialylated glycans and receptors likely play a key role in determining the tissue tropism of EV-D68. Sialic acid (a carbohydrate moiety) and intercellular adhesion molecule 5 (ICAM-5), a sialic acid containing glycoprotein, have both been identified as molecules which are bound by EV-D68 and facilitate viral entry (6–8). However, in a mouse model of EV-D68, the motor neurons which are infected and killed by the virus do not express detectable ICAM-5 levels, nor was detectable ICAM-5 expression found in adult or pediatric cervical spinal cord samples from humans (9), suggesting that it is unlikely ICAM-5 is responsible for viral entry into the spinal cord. Alternatively, sialylated glycans are ubiquitously expressed in the spinal cord, muscle, and lungs. But despite the strong dependence of some strains on sialic acid (7), other strains can infect cells in a sialic acid-independent manner (6), suggesting that sialic acid is not the only moiety to enable EV-D68 entry. Even among strains which require sialic acid, EV-D68 shows a large degree of promiscuity in the wide range of sialic acid linkages it can bind (10). It has been proposed that EV-D68 is capable of using multiple redundant receptors (6); this is consistent with the idea that EV-D68 can adhere to diverse binding molecules on an extracellular vesicle, which can then be taken into the host cell through various pathways.

Extracellular vesicles are lipid bilayer-enclosed, extracellular structures which can form by outward budding of the plasma membrane or by an intracellular endocytic trafficking pathway. The later involves the fusion of multivesicular bodies (MVBs) with the plasma membrane to release small extracellular vesicles, termed exosomes. Exosomes are less than 200 nm in diameter and can be differentiated from other small extracellular vesicles by the presence of the exosome-specific tetraspanins CD81 and CD63 (11). They are thought to be released by virtually all eukaryotic cells and can selectively deliver proteins, lipids, or genetic material to distant cells (12). Perhaps this ubiquitous vesicle could be coopted to shuttle virus between cells as well. Indeed, exosomal membranes richly expresses various adhesion molecules (13) and sialylated glycans (14). Some exosomes have even been shown to be enriched in α2,6-linked sialic acid (15), the specific linkage which is preferentially bound by EV-D68 (7). Transmission of multiple viral particles within extracellular vesicles has been reported in other enterovirus family members (poliovirus, coxsackievirus, and rhinovirus) (16), and this “en bloc” transmission has been shown to benefit viral replication and even increase neurovirulence (17, 18).

Here, we demonstrate that EV-D68 viral particles can adhere to the exterior surface of exosomes. Three peaks in viral RNA and viral titer were identified by ultracentrifugation of supernatant from EV-D68-infected cells through an isopycnic density gradient. The lowest-density peak, centered at 1.11 g/cm^3^, was characterized using various biochemical and structural techniques, including nanosight, electron microscopy, and immunoprecipitation; this was identified as exosome-associated EV-D68. The two higher-density peaks, centered at 1.20 and 1.24 g/cm^3^, were composed of infectious, RNA-containing viral particles that were not associated with exosomes. The viral form which equilibrated at 1.20 g/cm^3^ was found to have a 10- to 100-fold greater viral titer in contemporary EV-D68 clades (B1 and B, respectively) compared to the prototypic Fermon strain, suggesting that this form may have played a role in the recent surge in EV-D68-associated disease.

## RESULTS

### Infectious EV-D68 particles have three distinct densities.

**FIG 1:**
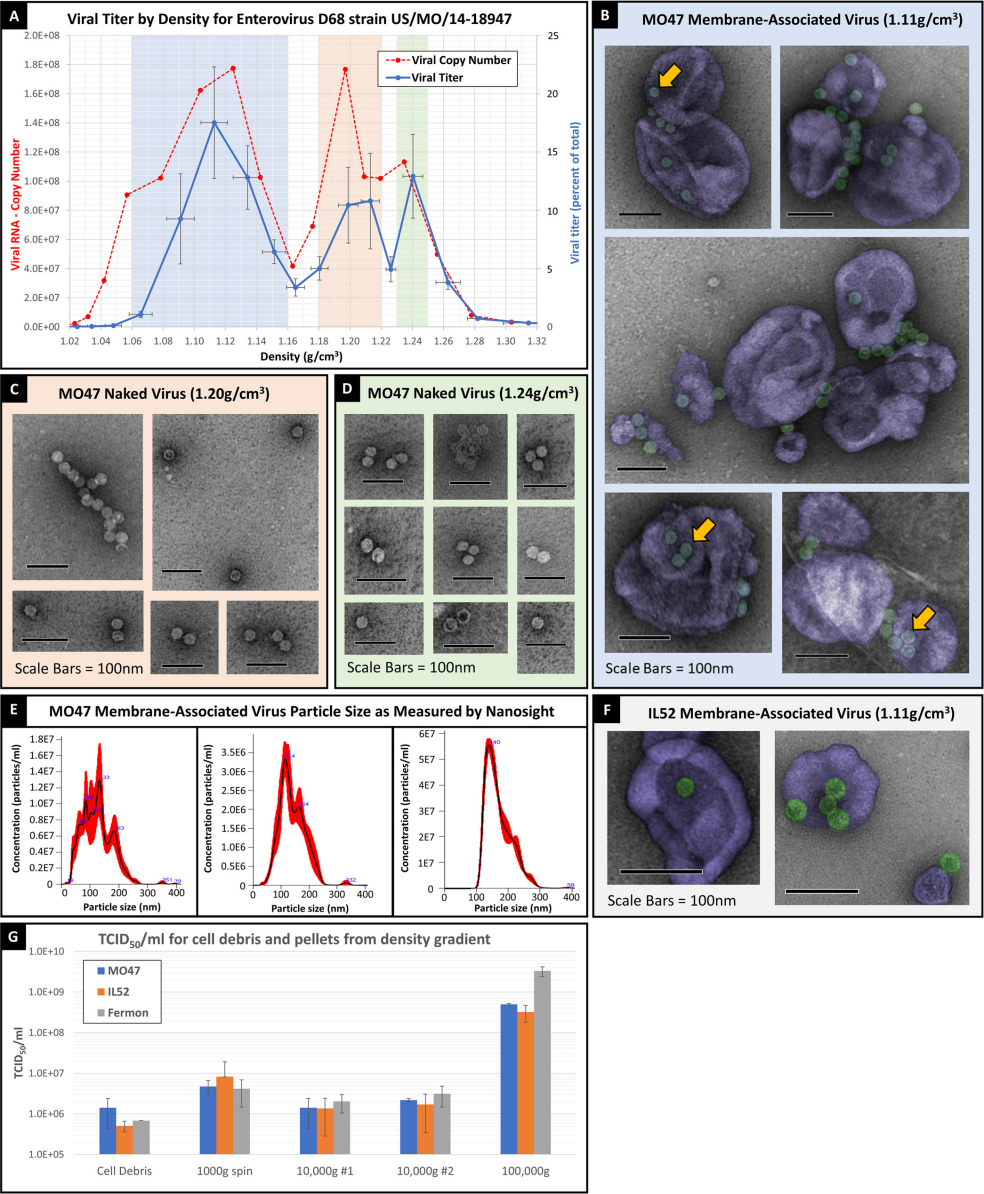
Three densities of EV-D68 (A) Density centrifugation of EV-D68 (strain US/MO/14-18947) showing viral copy number (dashed red line) and percentage of total viral titer (solid blue line) for densities between 1.02 and 1.32 g/cm^3^. “Viral titer: percentage of total” on right-hand *y* axis represents the TCID_50_ at each fraction divided by the total TCID_50_ for all fractions from the same sample. Error bars represent standard error of the mean (SEM) from 8 biological replicates. (B) False-color image of US/MO/14-18947 membrane-associated virus (1.11 g/cm^3^). Uranyl-formate stain followed by electron microscopy (blue highlight in panel A represents density range for membrane-associated virus). Yellow arrows point to viral particles associated with membrane. (C) Uranyl-formate stain followed by electron microscopy imaging of viral particles found at 1.20 g/cm^3^ density (panel A, orange highlight). (D) Uranyl-formate stain followed by electron microscopy imaging of viral particles found at 1.24 g/cm^3^ density (panel A, green highlight). (E) Nanosight measurements of particle size and concentration of membrane-associated virus (1.11 g/cm3) from US/MO/14-18947 (3 biological replicates). (F) False-color image of membrane-associated virus from RD cells infected with US/IL/14-18952. (G) TCID_50_/mL of cell debris and all pellets which were generated during density gradient protocol. Three biological replicates for each of the EV-D68 strains. Error bars represent standard deviation.

Rhabdomyosarcoma (RD) cells were infected with the prototypic Fermon strain of enterovirus D68 (EV-D68) as well as with two contemporary strains isolated from the 2014 outbreak, which have subsequently been shown to cause paralytic disease in mice: US/MO/14-18947 (B1 clade) and US/IL/14-18952 (B clade). Analysis of the US/MO/14-18947 (MO47) strain revealed three distinct peaks in viral titer (increased TCID_50_ [50% tissue culture infective dose]) and three commensurate peaks in viral RNA genome copy number (Fig. 1A). Electron microscopy (EM) of the broad, low-density peak, centered at 1.11 g/cm^3^, revealed that viral particles at this density appear to associate with membranous vesicles which are between 50 and 250 nm in diameter (Fig. 1B). We will thus refer to the virus found at this density as “membrane-associated virus.” Analysis of the membrane-associated viral fraction by Nanosight (which uses Brownian motion to estimate particle size) revealed that the average particle size was approximately 140 nm in diameter (Fig. 1E). Density centrifugation also identified two additional peaks in viral titer, centered at 1.20 and 1.24 g/cm^3^. Electron microscopy revealed “naked” (non-membrane-associated) viral particles at both of these densities (Fig. 1C and D); however, despite their differing densities, a visual analysis of EM images did not reveal any obvious differences between these two “naked” viral densities. Measurements of EV-D68 capsid diameter by EM revealed that the average EV-D68 capsid size was 32 ± 5 nm for all densities (including the viral particles which were associated with membrane), which is the expected capsid size for EV-D68. Electron microscopy of the 1.11 g/cm^3^ density of a different contemporary strain, US/IL/14-18952 (abbreviated “IL-52”), revealed that membrane-associated virus was present in this strain as well (Fig. 1F). We chose to focus our analysis on the 100,000-g pellet because, for all strains analyzed, we observed over 100-fold greater viral titers in this fraction compared to those of the cell debris, 1,000-g cell pellet, and 10,000-g cell pellets (Fig. 1G).

### Membrane-associated virus is composed of fully infectious EV-D68 viral particles that are associated with lipid membrane.

**FIG 2:**
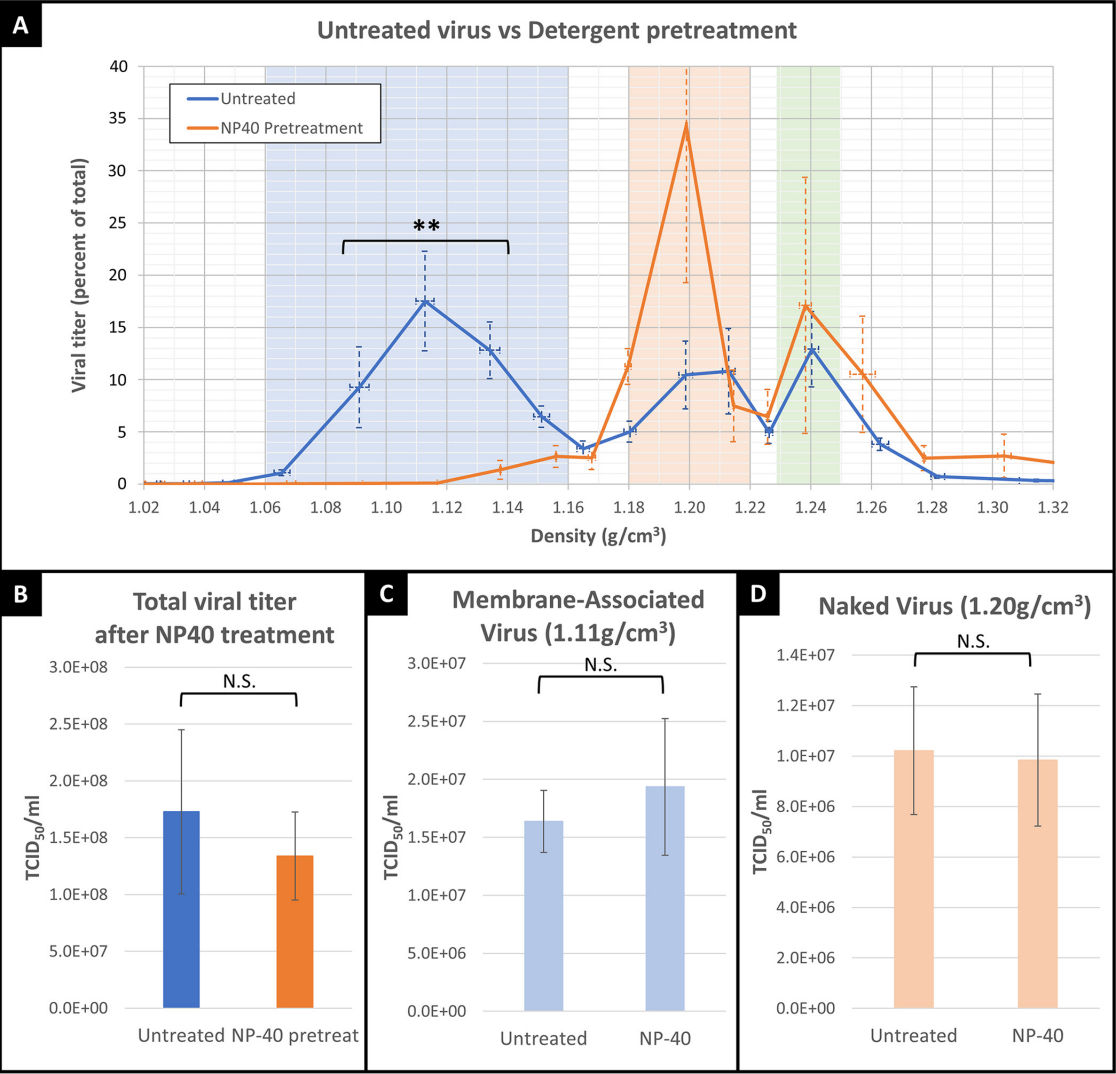
Treatment with nonionic detergent (NP-40) before or after centrifugation. (A) Blue line represents the viral titer of untreated EV-D68, orange line represents viral supernatant which was treated with nonionic detergent prior to density centrifugation. For virus density within the blue highlighted “membrane-associated virus” range (1.06 to 1.16 g/cm^3^), TCID_50_ values in NP-40-treated (orange line) samples are are significantly lower than those of untreated (blue line) samples (repeated measures analysis of variance [ANOVA]; **, *P* = 0.0037). (B) Total viral titer of supernatant (all densities combined) is not significantly decreased by NP-40 treatment. (C) Isolates of membrane-associated viral density (1.11 g/cm^3^) after NP-40 treatment. Viral titer was not significantly affected by NP-40 treatment. (D) Isolates of naked virus density (1.20 g/cm^3^) after NP-40 treatment. Viral titer was not significantly affected by NP-40 treatment. Error bars represent standard deviation in all figures.

Electron microscopy of the 1.11 g/cm^3^ density revealed viral particles which appeared to be closely associated with membranous vesicles (Fig. 1B). To determine whether the virus was associating with lipid membranes, we treated viral supernatant with nonionic detergent (NP-40) prior to density centrifugation. Detergent treatment of viral supernatant destroyed the lipid vesicles (confirmed by electron microscopy analysis; data not shown) and shifted the viral titer from the membranous peak, between 1.06 and 1.16 g/cm^3^, into the “naked” virus peaks at 1.20 g/cm^3^ and 1.24 g/cm^3^ (Fig. 2A), confirming that virus which equilibrated at 1.11 g/cm^3^ is attached to lipid membranes. Furthermore, the total viral titer (the sum of viral titers across all density fractions) for samples which were treated with NP-40 prior to centrifugation were the same as those of the untreated controls (Fig. 2B). This indicated that the viral titer was being shifted to a higher density but was not destroyed. To further confirm that NP-40 treatment did not reduce viral titer, we treated membrane-associated virus (Fig. 2C) and naked virus (Fig. 2D) with NP-40 and found no significant difference in TCID_50_ following treatment for either group. Thus, membrane-associated virus (with a density of 1.11 g/cm^3^) is composed of fully infectious EV-D68 viral particles (with an expected density of 1.20 or 1.24 g/cm^3^) that have associated with lipid membrane.

### Membrane-associated virus is not protected from immune recognition.

**FIG 3:**
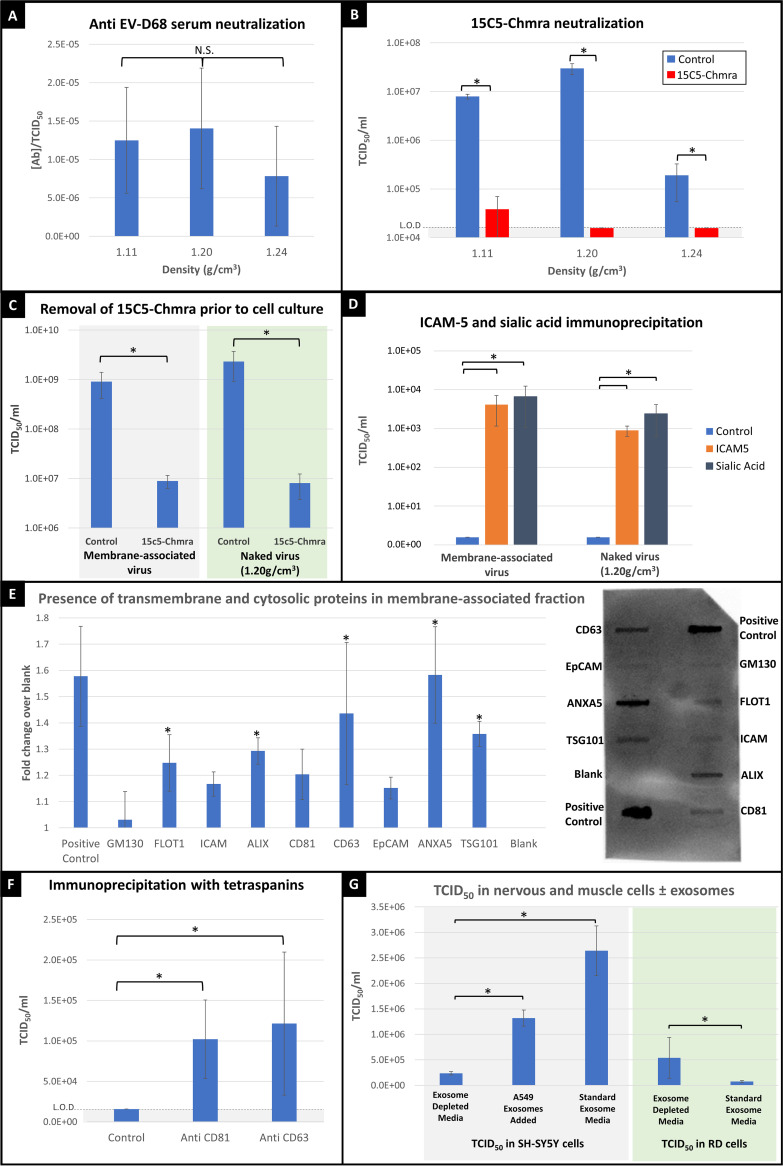
Immune recognition of various EV-D68 densities and characterization of membrane-associated virus. (A) The *y* axis represents average dilutions of anti-EV-D68 mouse serum required to neutralize virus, divided by the average TCID_50_ for respective viral densities (ANOVA *post hoc* Student'’s *t* test, *P* = 0.42, *P* = 0.68, *P* = 0.70). (B) Three viral density isolates (1.11, 1.20, and 1.24 g/cm^3^) were treated with 0.01 mg/mL 15C5-Chmra antibody for 1 h, then mix was put onto TCID_50_ plates to assess the viral titer of each isolate. Gray highlight represents detection limit. Asterisks (*) indicate statistical significance (1.11 g/cm^3^, *P* = 0.0003; 1.20 g/cm^3^, *P* < 0.0001; 1.24 g/cm^3^, *P* = 0.0064), all versus respective control, determined by Dunnett’s Method. (C) 15C5-Chmra antibody bound to magnetic beads was added to membrane-associated and naked virus. After 1 h, a magnet was used to remove antibody and the supernatant was added to a TCID_50_ plate to assess viral titer (15C5-Chmra versus control: *, *P* < 0.0005 for both membrane-associated and naked virus; Dunnett’s Method). (D) ICAM-5 or *N*-acetylneuraminic acid (sialic acid) were attached to magnetic beads and the antibody/bead complex was incubated with membrane-associated or naked virus samples for 1 h. Beads were rinsed twice in excess PBS and viral titer was assessed to determine how much virus was immunoprecipitated from the supernatant (control versus ICAM5 and control versus sialic acid for membrane-associated and naked virus; *, *P* = 0.0001 determined by Dunnett’s Method). (E) Exosome antibody array on 1.11 g/cm^3^ fraction, examining cytosolic proteins (FLOT1, ALIX, TSG101), transmembrane proteins (CD63, CD81, ANXA5), and *cis*-golgi matrix protein as markers for cellular contamination (GM130). Example blot is shown on the right and chart represents average intensity across three biological replicates. Positive control indicates detection reagents are working correctly, and do not represent an exosome-specific control. Error bars represent standard deviation. Statistics: comparison with control (blank) using Dunnett’s Method (*P* = 0.999 for GM130; *, *P* = 0.027 for FLOT1; *P* = 0.218 for ICAM; *, *P* = 0.005 for ALIX; *P* = 0.086 for CD81; *, *P* < 0.0001 for CD63; *P* = 0.305 for EpCAM; *, *P* < 0.0001 for ANXA5; *, *P* = 0.0008 for TSG101). Asterisks indicate statistical significance. (F) Anti-CD81 or anti-CD63 antibodies were attached to magnetic beads and incubated with membrane-associated virus. Supernatant was discarded, and beads were rinsed and treated with 0.01% NP-40 (to dissolve exosomes and release virus from bead) before TCID_50_ measurement. CD81 versus control: *, *P* = 0.0178; CD63 versus control: *, *P* = 0.0180 as determined by Dunnett’s Method. Gray highlight represents detection limit. (G) RD or SH-SY5Y cells in TCID_50_ plate were infected with MO47 with or without exosomes in the medium. The “A549 exosomes added” bar represents exosome-depleted media to which purified A549 exosomes were added. Gray panel represents TCID_50_ plates containing SH-SY5Y cells. Each condition represents 3 biological replicates. Error bars represent standard deviation. ANOVA: *, *P* < 0.05. Green panel represents TCID_50_ plates containing RD cells. Each condition represents 4 biological replicates. Error bars represent standard deviation. ANOVA: *, *P* < 0.05.

Since other picornaviruses are known to utilize lipid membranes to “hide” from the immune system (19), we next tested whether anti-EV-D68 immune serum (from mice inoculated with US/MO/14-18947, abbreviated “MO47”) was able to neutralize both naked and membrane-bound virus with the same efficiency. We found that all three viral densities of EV-D68 strain MO47 were neutralized by equivalent dilutions of anti-EV-D68 mouse serum (Fig. 3A). All densities were also neutralized by equivalent dilutions of human intravenous immunoglobulin (IVIg) (adult humans have nearly complete seroprevalence of neutralizing antibodies for EV-D68; data not shown). These results indicate that the membranous vesicles do not protect the virus from immune recognition or neutralization.

Since it is difficult to envision how viral particles which are completely surrounded by membrane could be neutralized by antibody, we next tested whether viral particles were adhering to the outside of the lipid membrane. Membrane-associated virus was isolated by ultracentrifugation and exposed to a chimeric mouse/human monoclonal antibody which targets the viral capsid at the 3-fold axis. This antibody, referred to as “15C5-Chmra,” has the same F_ab_ fragment as described by Zheng et al. (20), but the F_c_ fragment is human. The15C5-Chmra antibody neutralized virus from all three density isolates (Fig. 3B). This finding is consistent with the hypothesis that membrane-associated viral particles are found on the exterior of the membrane rather than hiding on the inside of it.

To address the possibility that 15C5-Chmra antibody was neutralizing the virus during the short interval after release from the vesicle, but prior to viral entry into the cell; we covalently attached 15C5-Chmra antibody to magnetic beads so that the antibody could be removed prior to adding the virus to cell culture. We found that 15C5-Chmra antibody neutralized both membrane-associated and naked virus, even if antibody was removed prior to placing virus onto cells (Fig. 3C). Taken together, we conclude that the membrane-associated viral fraction is composed of EV-D68 virions that have attached to the exterior surface of membranes.

### EV-D68 can adhere to beads coated with ICAM-5 and sialic acid.

To further examine what surface molecules might permit EV-D68 to attach to these membranes, we covalently attached intercellular adhesion molecule 5 (ICAM-5) or sialic acid (*N*-acetylneuraminic acid) to magnetic beads and used these known EV-D68-binding molecules to immunoprecipitate virus from the supernatant. We found that both ICAM-5 and sialic acid immunoprecipitated approximately 1,000-fold more viral titer from supernatant than the control (nonspecific antibody-coated beads), confirming that EV-D68 can bind to cellular surfaces expressing ICAM-5 or sialic acid (Fig. 3D) and does so with a high enough affinity to remain attached through multiple washes. This further demonstrates that virus remains infectious after binding ICAM-5 or sialic acid and suggests that the binding is transient (since it is likely that the virus must detach from beads to infect cells in the TCID_50_ plate).

### Membrane-associated viral fraction contains viral particles attached to exosomes.

The vesicles that are associating with EV-D68 are consistent with exosomes in both density (11) and size. However, to address the possibility that these “vesicles” were fragments of either parent cell membrane or replication organelles, we examined whether the membrane-associated viral fraction contained cytosolic proteins (which are only present inside intact vesicles) and looked for a marker of Golgi membrane (indicating cellular contamination and the presence of replication organelles [21]). We found that various cytosolic proteins (FLOT1, ALIX, and TSG101) were present in the membrane-associated viral fraction, demonstrating that this fraction contains a significant number of intact vesicles (Fig. 3E). We further showed that these vesicles contain at least two transmembrane proteins (ANXA5 and CD63), demonstrating that the membrane is composed of a lipid bilayer (Fig. 3E). When we looked for the presence of GM130 (a *cis*-golgi matrix protein) in the membrane-associated viral fraction to assess contamination levels, we found that GM130 was virtually undetectable (Fig. 3E). We next examined whether exosome-specific antibodies (CD63- or CD81-) could immunoprecipitate virus from the supernatant of MO47-infected RD cells and found that CD63- and CD81-immunoprecipitated material contained significantly higher viral titers than the controls (Fig. 3F). Taking these findings together, we conclude that EV-D68 is capable of binding to the exterior surface of intact vesicles which have a lipid bilayer and express the exosome-specific tetraspanins CD63 and CD81.

Based on these data, we next asked whether exosomes play an important role in viral entry into permissive cells. To test this, we measured viral titers in the SH-SY5Y neuroblastoma cell line and in the RD rhabdomyosarcoma cell line, in the presence or absence of exosomes. We found that in the SH-SY5Y neuroblastoma cell line, viral titer was significantly reduced if exosomes were depleted from the medium prior to adding virus (Fig. 3G). When we supplemented the exosome-depleted medium with exosomes from a permissive lung epithelial cell line (A549 cell exosomes), the reduced viral titer was partially rescued (Fig. 3G), showing that exosomes play an important role in viral infection of a neuronal cell line. Conversely, when a permissive muscle cell line (RD cell line) was infected with EV-D68, the viral titer was significantly increased in the absence of exosomes (Fig. 3G), suggesting that exosomes do not play an important role in viral entry into muscle cells. We interpret these results to mean that EV-D68 preferentially infects RD cells by directly binding with the parent cell membrane (without needing to bind exosomes first), and that the decreased viral titer in exosome-containing medium can be attributed to adsorption of EV-D68 viral particles by the exosomes in standard medium (thereby sequestering virus away from parent cell membrane). It should be noted that we are not trying to make the case that RD cells do not take up exosomes, only that there is a direct pathway for viral uptake that does not rely upon exosomes. This direct uptake of virus is likely faster than the exosome-associated pathway, as indicated by the presence of ubiquitous cytopathic effects (CPE) by 7 days postinfection in RD cells. In SH-SY5Y neuroblastoma cells, we interpret these results to mean that exosomes do play an important role in viral entry and that binding to exosomes in the medium facilitates entry into cells. This exosome-associated infection pathway is likely slower than direct viral uptake, as indicated by the slow development of CPE over the course of 28 days in SH-SY5Y cells.

### Three distinct particle densities seen in prototypic and contemporary strains.

**FIG 4:**
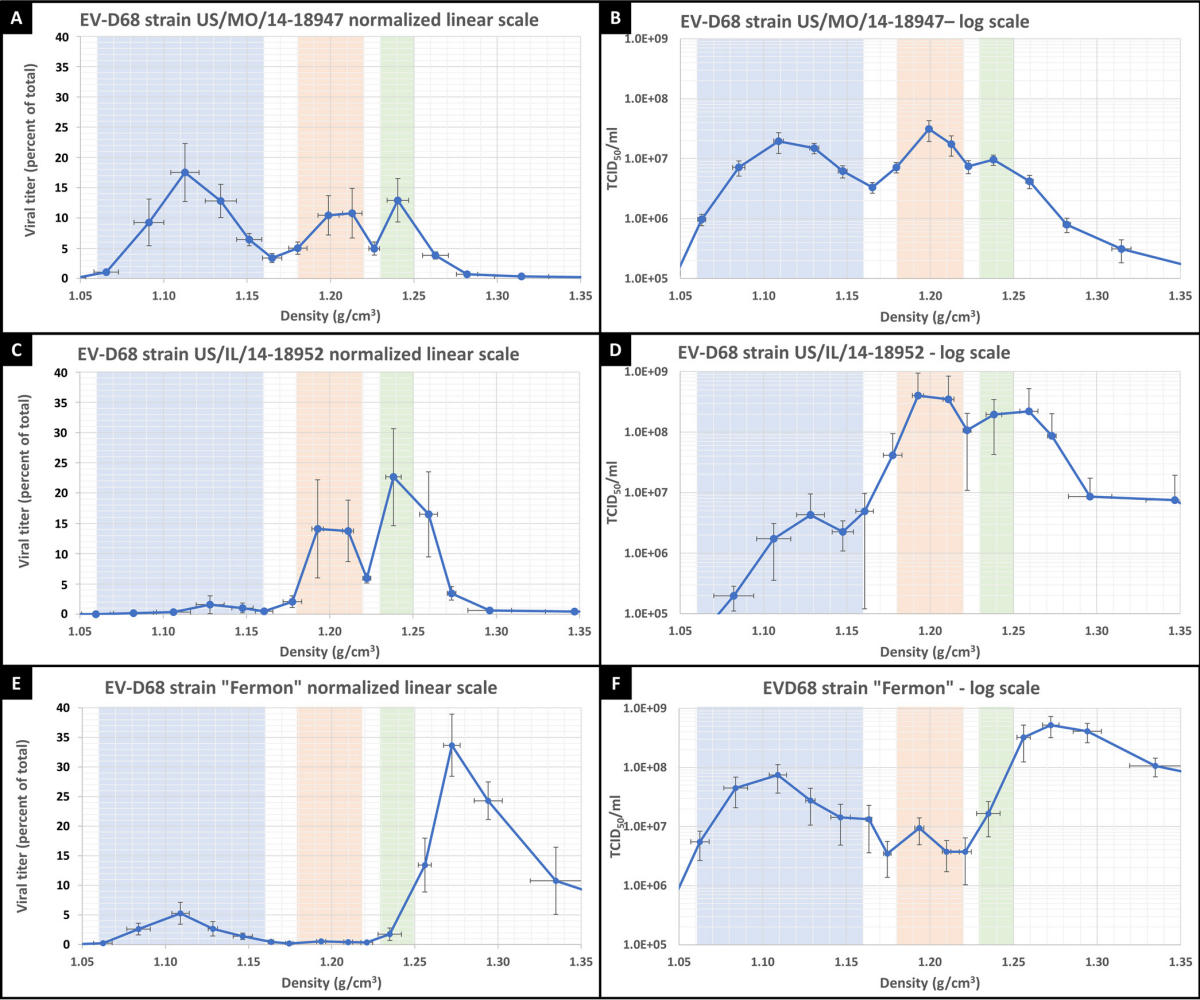
Viral titers of various EV-D68 strains at densities between 1.05 and 1.35 g/cm^3^, with blue, orange, and green highlights standardized to “US/MO/14-18947 grown in RD cells” to facilitate comparison between strains and cell lines. The *y* axis in panels A, C, and E, labeled “Viral titer (percentage of total),” represents the TCID_50_ at each fraction divided by the summed TCID_50_ for all fractions from the same sample (this normalizes for differences in overall viral titer between samples), and data are plotted on a linear scale. The *y* axis in panels B, D, and F represents direct TCID_50_ measurements (non-normalized) and is plotted on a logarithmic scale. (A) EV-D68 strain US/MO/14-18947 grown in rhabdomyosarcoma (RD) cells. Error bars represent SEM across 8 biological replicates. (B) Same data as in panel A, but plotted on a log scale and non-normalized. (C) EV-D68 strain US/IL/14-18952 grown in RD cells. Error bars represent SEM across 5 biological replicates. (D) Same data as in panel C, but plotted on a log scale and non-normalized. (E) EV-D68 ancestral Fermon strain grown in RD cells. Error bars represent SEM across 8 biological replicates. (F) Same data as in panel E, but plotted on a log scale and non-normalized.

We further compared the viral titers of two contemporary EV-D68 strains (MO47 and IL-52) and one prototypic strain (Fermon) and found differences in the magnitude of the exosome-associated peak, as well as differences in the magnitudes of the denser “naked” viral peaks. Figures 4A, C, and E show the normalized viral titers of strains MO47, IL-52, and Fermon, respectively, on a linear scale. For comparison, Fig. 4B, D, and F show the raw TCID_50_ data for the same strains on a logarithmic scale. All of the EV-D68 strains we examined gave rise to three distinct particle densities, but with notable differences in the magnitude of viral titers when comparing equivalent densities between strains. Exosome-associated virus (blue highlight) was seen in all strains but was most evident in MO47, where it represents about 45% of total viral titer (Fig. 4A), compared to approximately 3% and 12% for IL-52 (Fig. 4C) and Fermon (Fig. 4E), respectively. Despite the low viral titers seen in the exosome-associated peak for IL-52 (Fig. 4C), electron microscopy revealed IL-52 viral particles bound to small vesicles (Fig. 1F), and RD cells infected with the IL-52 or Fermon strain in exosome-depleted media showed the same (approximately 10-fold) increase in viral titer as MO47 (data not shown), indicating that the IL-52 and Fermon strains just are capable of binding exosomes as MO47. We suggest that variations in binding kinetics or receptor affinity between EV-D68 strains could affect the stability of viral association with exosomes and impact the amount of membrane-associated virus that remains bound to exosomes throughout centrifugation. We further conclude that association with exosomes is a ubiquitous EV-D68 characteristic which is present in both the prototypic and modern strains.

### Non-exosome-associated fraction (at 1.20 g/cm^3^) has higher viral titer in 2014 EV-D68 strains.

Examination of the denser, non-exosome-associated viral particles revealed significant differences between the modern strains and the prototypic strain in the viral peak centered at 1.20 g/cm^3^. Since viral particles at this density are not associated with exosomes, differential binding kinetics and dissociation from lipid vesicles during centrifugation cannot explain the reduced viral titer we observed in the Fermon strain at this density. We found that less than 1% of the total viral titer for the prototypic Fermon strain was found within the orange highlighted region (between 1.18 and 1.22 g/cm^3^), whereas approximately 22% and 28% of the viral titer was found in this density range for the contemporary EV-D68 strains (2014 isolates) MO47 and IL-52, respectively (Fig. 4A, C, and E, orange highlight). To address the possibility that the reduced viral titer of the Fermon strain at 1.20 g/cm^3^ was due to a greater loss of viral particles in the discarded cell debris or pellets, we measured the viral titers of discarded cell debris and all discarded pellets and found no difference in the viral titers of discarded material between any of the strains (Fig. 1G). Furthermore, if viral particles were being specifically lost from the Fermon strain, we would expect to see a reduction in the total viral titer (cell debris plus all pellets) of this strain; however, the total viral titer of the Fermon strain was actually slightly higher than that of either IL-52 or MO47 (Fig. 1G). Nevertheless, it appears that the 1.20 g/cm^3^ particle density is present in the Fermon strain when the data is viewed in a log scale, albeit at a very low titer (Fig. 4F, orange highlight). We thus conclude that the 1.20 g/cm^3^ particle density has a much greater viral titer in the modern EV-D68 strains than in the prototypic Fermon strain.

### Analysis of non-exosome-associated viral particles at densities above 1.24 g/cm^3^.

**FIG 5:**
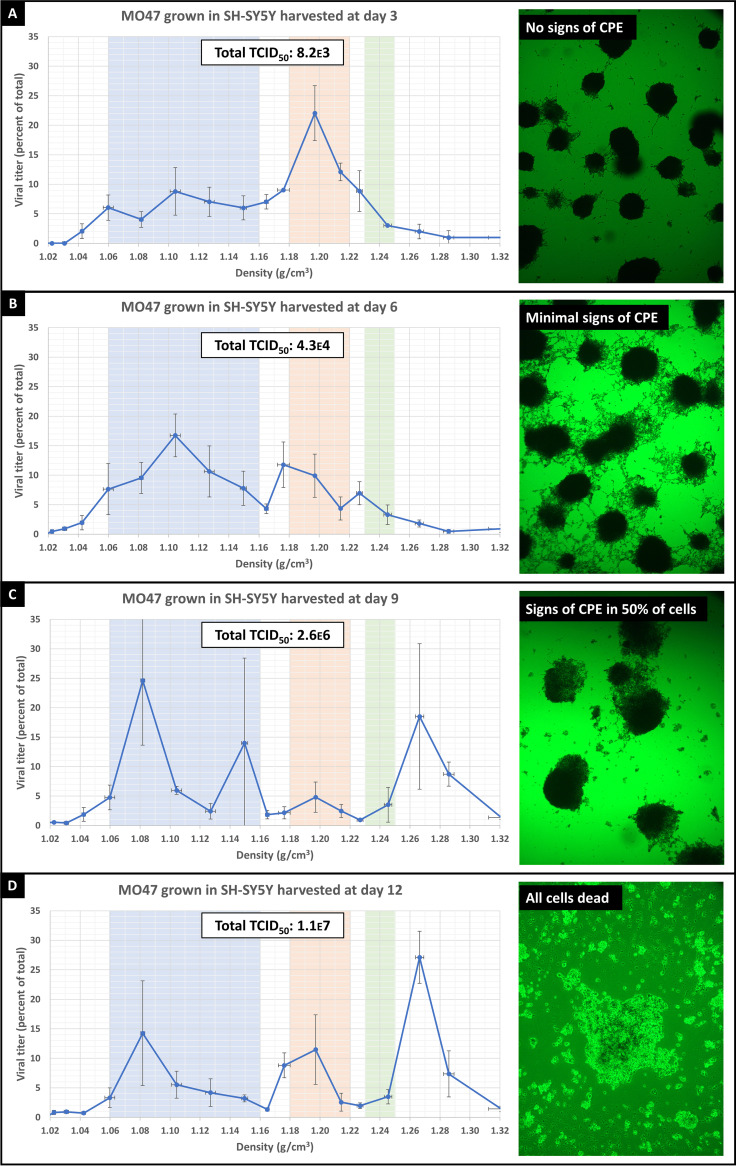
Each graph (A to D) represents viral titers of supernatant collected from US/MO/14-18947 grown in a neuroblastoma cell line (SH-SY5Y) and collected at four consecutive time points at 3-day intervals (day 3, 6, 9, and 12). The *y* axis represents the TCID_50_ at each density divided by the total TCID_50_ for the whole sample. Error bars represent SEM across 3 biological replicates. Average “total TCID_50_” viral titers for each time point are shown in the box at the top of each chart. To the right of each graph is a representative image of virus-infected SH-SY5Y cells for which viability was assessed using an MTT [3-(4,5-dimethylthiazol-2-yl)-2,5-diphenyltetrazolium bromide] assay. Metabolically active cells turn dark blue in the presence of MTT.

We also observed that the highest density peak in the prototypic Fermon strain was increased to 1.27 g/cm^3^, up from the expected density of 1.24 g/cm^3^ (Fig. 4E, green highlight). We do not know why the highest density peak was increased in the Fermon strain. However, we saw the same shift in the highest density peak of the EV-D68 strain MO47 when supernatant from infected RD cells was compared to that from infected SH-SY5Y cells (Fig. 5D), suggesting that the density of this form of EV-D68 can vary when the virus is grown in different cell types.

### Viral particles released from EV-D68-infected SH-SY5Y neuroblastoma cell line.

To test whether the three densities of EV-D68 particles which we observed in RD cells was specific to that cell type, we next analyzed virus grown in SH-SY5Y neuroblastoma cells. Infected SH-SY5Y cells took much longer to show signs of cytopathic effects, and ≥95% cell death was not observed until 12 days after initial infection (compared to 3 days for RD cells) (Fig. 5A to C). As in RD cells, we found that three distinct forms of EV-D68 are released into the supernatant of infected SH-SY5Y cells (Fig. 5D). We took advantage of the significantly longer time to CPE and cell death in SH-SY5Y cells to examine whether certain viral forms are preferentially released at earlier time points during infection. In general, the membrane-associated viral form and the naked viral form found at 1.20 g/cm^3^ were released earlier in the infection (Fig. 5A–D), with the highest-density, non-exosome-associated form (found at 1.24 to 1.27 g/cm^3^) showing up only after there were obvious signs of cell death in a significant portion of host cells (Fig. 5C–D).

## DISCUSSION

### Discussion of exosome-associated viral peak at 1.11 g/cm^3^.

Here, we show that EV-D68 grown in both muscle and neuronal cell lines gives rise to three distinct densities of viral particles. The least-dense fraction was composed of EV-D68 particles which had attached to the exterior surface of a specific type of small extracellular vesicle called an exosome. We found that viral particles are infectious in this exosome-associated form and that associating with exosomes plays an important role in viral infection of a neuronal cell line. While most of the characterization of exosome-associated virus was performed in the EV-D68 strain US/MO/14-18947 (MO47), we found evidence that another contemporary strain, US/IL/14-18952 (IL-52), and the prototypic Fermon strain can bind exosomes as well, suggesting that association with exosomes is a ubiquitous EV-D68 characteristic.

### EV-D68 binding to exosomes and implications on CNS entry and receptors.

EV-D68 is capable of binding both sialic acid- and ICAM-5 coated beads through multiple rinse steps while retaining its ability to detach and infect cells (Fig. 3D), suggesting that virus can bind to vesicles which express either of these molecules. Sialylated glycans are richly expressed on the surface of exosomes. We hypothesize that EV-D68 can utilize various attachment factors, such as sialylated glycans and adhesion molecules, to attach to the surface of exosomes. In fact, various sialic acid-binding lectins show an even greater affinity for exosomes than for parent cell membrane (14). Our data indicate that EV-D68 can bind to exosomes in cell media, which facilitates infection of neural SH-SY5Y cells and interferes with infection of muscle (RD) cells (Fig. 3G). In the neural SH-SY5Y cell line, EV-D68 particles produced a higher viral titer when exosomes were present in the medium, suggesting that EV-D68 may gain entry to neural cells when infected exosomes are taken into the cell. Conversely, in the RD cell line, EV-D68 particles produced a lower viral titer when exosomes were present in the medium, suggesting that the majority of viral titer results from free viral particles which bind directly to the muscle cell membrane, and that the presence of exosomes in the media sequesters virus away from parent cell membrane. Our data suggest that EV-D68 can gain access to the cell directly by binding with the cell membrane or indirectly by binding exosomes (which are subsequently taken into the cell), which fits well with the wide range of sialic acid and ICAM binding partners that EV-D68 can utilize.

A haploid genetic screen of the prototypic Fermon strain identified several genes involved in the biosynthesis, transport, and conjugation of sialic acid which are essential for infection, but it failed to identify any specific protein receptor, leading the authors to conclude that EVD68 may be able to use multiple, redundant sialylated receptors (6). If EV-D68 can utilize various sialylated proteins, glycans, and intracellular adhesion molecules as attachment factors on exosomes, there would be tremendous variability and redundancy in the EV-D68 receptor; and this would explain why a single EV-D68 receptor has not yet been identified. Furthermore, we hypothesize that differences in binding affinity and/or kinetics between virus and attachment factor may account for the variability in the size of the membrane associated peak shown in Fig. 4A, C, and E. We would expect that the viral titer of exosome-associated virus might differ between strains, and even within the same strain grown in different cell types, if the exosomes released by those cell types have different attachment factors.

### Does EV-D68 benefit from associating with exosomes?

EV-D68 is similar to other picornaviruses in its ability to associate with lipid vesicles, but dissimilar in that EV-D68 is attaching to the exterior of the vesicles rather than hiding inside them (19, 22). For this reason, membrane-associated EV-D68 is not protected from immune recognition or neutralizing antibodies (Fig. 3A, B, and C). However, protection from host immune factors is not the only possible benefit of associating with vesicles. Several members of the enterovirus family (poliovirus, coxsackievirus, and rhinovirus) have been reported to exit cells in large numbers inside extracellular vesicles (16), and this “en bloc” transmission has been shown to benefit viral replication within the cell and even increase neurovirulence (17, 18). This suggests that association of multiple EV-D68 virions with a single exosome may have similar benefits if virally contaminated exosomes gain entry to uninfected host cells or if virally contaminated exosomes are swept up the respiratory tract and aerosolized to infect the next host with a package of EV-D68 particles. If this turns out to be the case, it suggests that infected exosomes may accidentally facilitate the shuttling of virus from lungs to spinal cord in an infected host, even in the absence of detectable viremia. However, infection of the CNS is unlikely to benefit EV-D68 since the virus does not establish latency in motor neurons nor is it able to spread to new hosts through CNS infection.

### Does EV-D68 adhere to exosomes before or after release?

**FIG 6:**
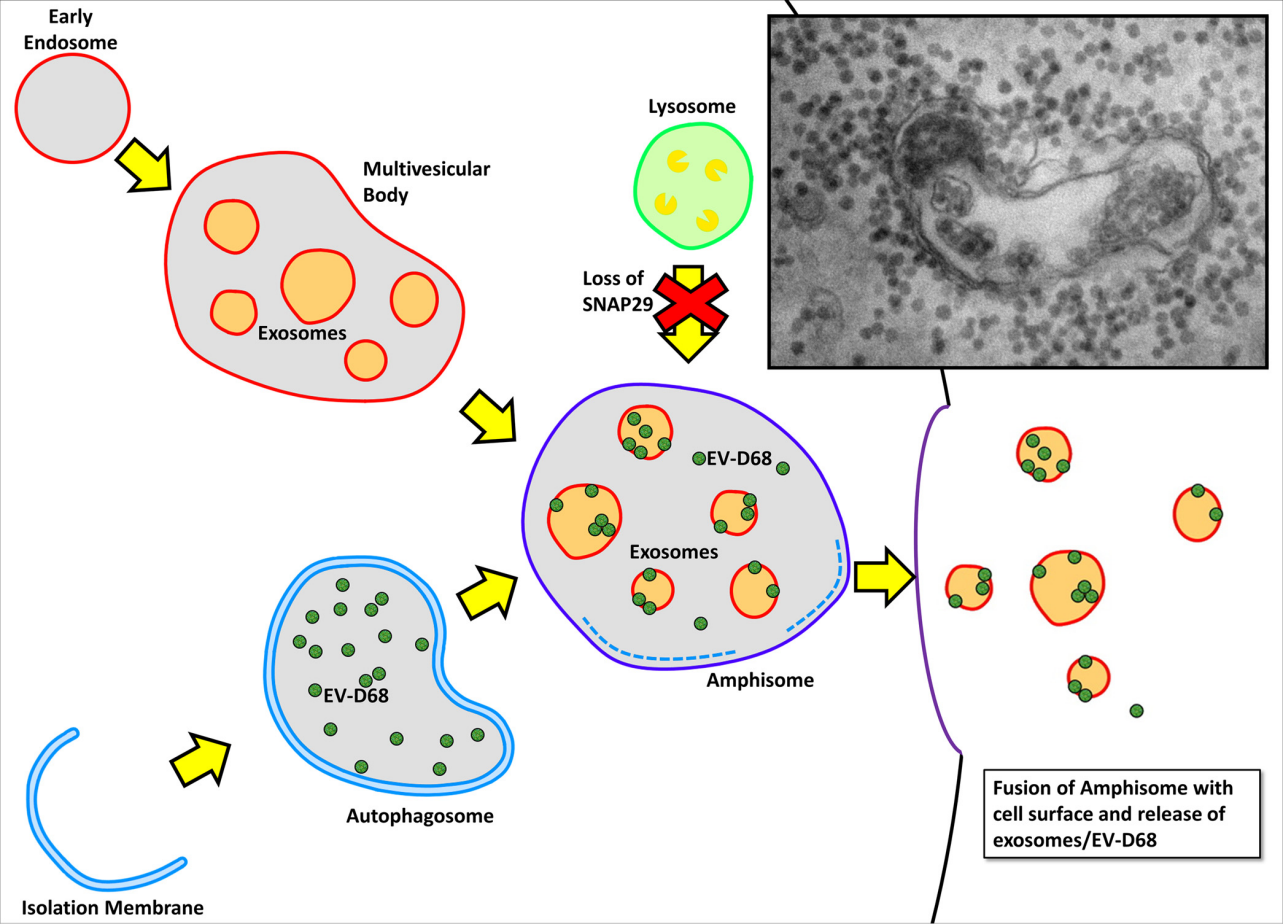
Schematic showing the hypothesized mechanism of exosome-associated EV-D68 release from host cell. Fusion of the autophagosome (containing nascent EV-D68 viral particles) with the multivesicular body (containing nascent exosomes) forms the amphisome where EV-D68 particles mature. Loss of SNAP29 prevents degradation of EV-D68 inside the amphisome and likely encourages extracellular release of amphisomal contents. This figure is adapted from Corona et al. (23) to include MVBs and exosomes. Inset shows an electron micrograph of a structure which appears to be an amphisome containing exosome-associated EV-D68 (electron microscopy image of EV-D68-infected SH-SY5Y cells).

We observed multiple EV-D68 viral particles attached to a single exosome (Fig. 1B), which is a requirement for the increased replication efficiency seen in other vesicle-enclosed picornaviruses. Our work shows that it is possible for viral particles to attach to exosomes in the surrounding medium and that the rupture of infected cells *in vivo* might inundate any nearby exosomes with virus; however, work by Corona et al. (23, 24) suggests another intriguing possibility. Corona et al. showed that the EV-D68-encoded protease 3C cleaves the membrane fusion protein SNAP-29 in order to facilitate viral replication, and they further hypothesized that this fusion generates a dominate negative fragment which interferes with fusion of the amphisome and lysosome, preventing viral degradation. This, in turn, leads to the accumulation of vesicles within the cell, believed to be either autophagosomes or amphisomes. If these vesicles are indeed amphisomes, then they represent the ideal environment for EV-D68 to bind exosomes prior to extracellular release. The amphisome represents a fusion between the autophagosome (containing nascent EV-D68 virions [25]) and endocytic multivesicular bodies (MVB; 26) (containing intraluminal vesicles which are renamed exosomes upon extracellular release [27]) (Fig. 6). Moreover, acidification of the amphisome is thought to play an important role in EV-D68 maturation (25), suggesting that EV-D68 particles are likely to be maturing right alongside exosomes, within the same MVB. Fusion of the amphisome with the cell membrane would allow extracellular release of virus and exosomes without cell lysis (similar to that of other picornaviruses which hijack the autophagy pathway). This is consistent with our finding that the 1.11 g/cm^3^ (exosome-associated virus) and 1.20 g/cm^3^ viral densities appear in the supernatant many days before any signs of cytopathic effect or cell death (Fig. 5A).

### Differences between strains in non-exosome-associated viral peak at 1.20 g/cm^3^.

A key difference between the contemporary and prototypic EV-D68 strains we examined was seen in the non-exosome-associated viral peak found at a density of 1.20 ± 0.02 g/cm^3^. This density represents approximately 22% and 28% of total viral titer for the MO47 and IL-52 strains, respectively. In contrast, less than 1% of the total viral titer for the ancestral Fermon strain is found at this density (Fig. 4A, C, and D). These differences cannot be explained by differential binding kinetics or dissociation from exosomes during centrifugation, as we suggest is the explanation for differences seen in the 1.11 g/cm^3^ (exosome-associated) density. There is amassing evidence that the prevalence of EV-D68 cases has dramatically increased during the latter half of the 20th century, with the first major outbreak in the United States occurring in 2014 (1), followed by subsequent outbreaks in 2016 and 2018. Our finding that the 1.20 g/cm^3^ density has approximately 10- to 100-fold greater viral titer in 2014 isolates compared to the 1962 Fermon strain suggests that the viral particle at this density could have played a role in enabling the increasing prevalence of EV-D68 leading up to the 2014 outbreak. This, of course, raises the following question: “What viral particle is equilibrating at 1.20 g/cm^3^ density, and how is it different from the particle found at 1.24 g/cm^3^?” In our estimation, the most likely answer is that one density represents mature viral particles and the other represents A-particles (20). Since the mature virion is expected to be more compact than the A-particle, with average diameters along the 5-fold axis of 320 Å and 330 Å, respectively (20), we hypothesize that the 1.24 g/cm^3^ density is composed of mature viral particles and that the 1.20 g/cm^3^ density is composed of A-particles. Unfortunately, the standard deviation within our measured EV-D68 capsid size (taken via electron microscopy) was too large for us to differentiate between particles with diameters that differ by only 1 nm, so we cannot yet confirm the identity of this particle density. This is an area of ongoing research.

## MATERIALS AND METHODS

### Virus preparation.

Three strains of enterovirus D68 were obtained: US/MO/14-18947 (purchased from BEI Resources, NR-49129, lot no. 63205984), US/IL/14-18952 (purchased from BEI Resources, NR-49131, lot no. 63205985), and EV-D68 Fermon strain (generous gift from Charles Y. Chiu at the University of California, San Francisco, CA). Enterovirus D68 strains were grown in human muscle rhabdomyosarcoma (RD) cells (purchased from ATCC CCL-136) for all experiments except those shown in Fig. 5, for which US/MO/14-18947 was grown in SH-SY5Y neuroblastoma cells (purchased from ATCC CRL-2266). Virus was added at a multiplicity of infection (MOI) of 100 to RD cells in a T182 flask for 60 min, followed by a phosphate-buffered saline (PBS) rinse and incubation in 10 mL medium (95% high-glucose Dulbecco’s modified eagle medium [DMEM] + 5% fetal bovine serum [FBS]) for 3 days at 33°C. All strains killed 99% of RD cells in the flask after 3 days.

### Protocol for tissue culture infectious dose assay.

RD cells were plated at 3,000 cells/well into 2 sets of 5 columns in a 96-well, flat-bottomed cell culture plate. After 24 to 48 h, serial 10-fold dilutions of EV-D68 were added to the appropriate wells and incubated at 33°C for 7 days. All wells were examined for signs of cytopathic effect and 50% tissue culture infectious dose (TCID_50_) values were calculated using the Reed-Muench method. All TCID_50_ values in this paper represent TCID_50_/mL, even if it is not explicitly stated.

### Gradient centrifugation.

A Beckman Coulter L8-55M Ultracentrifuge with a SW-41 swinging bucket rotor was used for all high-speed centrifugation steps. Supernatant was harvested from culture flasks, centrifuged for 10 min at 1,000 × *g* (pellet discarded), centrifuged twice for 30 min at 10,000 × *g* (pellets discarded), and centrifuged for 60 min at 100,000 × *g*. Pellet was collected and separated on an isotonic (sucrose was the osmotic balancer) OptiPrep density gradient (20%, 30%, 40%, 50% OptiPrep). Density gradient was spun at 141,000 × *g* for 60 h, and 500-μL aliquotes were collected from the top (fraction no. 1) to the bottom (fraction no. 20) of the column. The refractive index, viral titer, and viral copy number for each fraction were experimentally determined using a refractometer, TCID_50_ assay, and real time PCR respectively.

### Real-time PCR protocol.

Viral RNA was isolated from each fraction using a QIAmp Viral RNA Mini Kit (cat no. 52904). The RT-PCR setup used reagents and master mix from the Bio-Rad iTaq Universal SYBR Green One-Step Kit (cat no. 1725150). Primer sequences were Fwd1-CAC(T/C)GAACCAGA(A/G)GAAGCCA, Rev1-CCAAAGCTGCTCTACTGAGAAA, and Rev2-CTAAAGCTGCCCTACTAAG(G/A)AA, as published by WashU (28). Thermal cycling conditions were 50°C for 10 min, 95°C for 2 min, then 40 cycles of 95°C for 15 sec and 60°C for 45 sec, followed by a melt curve analysis from 65 to 95°C in 0.5°C-increments. All samples had 3× technical replicates.

### Exosome characterization.

Three biological replicates of the exosome-associated viral fraction were diluted in 10 mL PBS and centrifuged at 100,000 × *g* for 90 min, then the supernatant was removed, and pellets were resuspended in PBS (to remove OptiPrep solution). The protein concentration was measured (∼5 mg/mL) and samples were prepared as described in the user manual for the “Exo-Check Exosome Antibody Array” (cat no. EXORAY210B) produced by System Biosciences. Antibody arrays were imaged using a Thermo Fisher iBright CL1500 Imaging System.

### Antibody and magnetic beads for immunoprecipitation.

We followed the coupling protocol from the Dynabeads Antibody Coupling Kit (Thermo Fisher, cat no. 14311D) to covalently attach magnetic beads to the following antibodies: anti-CD81 (1D6) monoclonal antibody (Novus Biologicals NB100-65805), anti-CD63 (H5C6) monoclonal antibody (Novus Biologicals NBP2-42225), 15C5-chimeric monoclonal antibody (generous gift from Michael Pauly at ZabBio), and anti-HSV negative control (generous gift from Michael Pauly at ZabBio). Antibody concentration was 30 μg Ab per mg beads.

### Anti-EV-D68 serum neutralization.

US/MO/14-18947 was grown in RD cells and the viral supernatant (from three biological replicates) was harvested after 3 days. Fractions were isolated as described previously and TCID_50_ was calculated for the 1.11, 1.20, and 1.24 g/cm^3^ densities. Each of these viral densities were then plated in a 96-well plate at both 1,000 TCID_50_ and 100 TCID_50_. Anti-EV-D68 mouse serum was serially diluted 2-fold (from 1:20 to 1:20,480) and mixed with virus in appropriate wells. Four technical replicates were assessed for both virus dilutions, and the average of the four replicates was calculated. The average concentration of anti-EV-D68 mouse serum necessary to prevent CPE was then divided by the measured TCID_50_ of the viral supernatant.

### ICAM-5 and sialic acid immunoprecipitation.

Dynabeads Antibody Coupling Kit protocol was used to attach sialic acid (Sigma A0812) or ICAM-5 (recombinant human ICAM-5 Fc Chimera 1950-M5-050) to magnetic bead resin (Thermo Fisher cat no. 14311D). Antibody/bead complex was incubated with the 1.11 or 1.20 g/cm^3^ viral densities for 1 h at 33°C, then overnight at 4°C. A magnet was used to separate the bead complex from the supernatant (supernatant was discarded). The magnetic beads plus any bound virus (approximately 1 mg beads per sample) were rinsed 2× for 10 min in 1,000 μL sterile PBS, with agitation. The rinsed beads (plus any bound virus) were then mixed with 180 μL of PBS plus 0.1% NP-40 and agitated for 5 min before the beads and supernatant were added to TCID_50_ plates for viral titer analysis.

### Mouse immune serum.

Mice were intramuscularly injected with US/MO/14-18947 at 1,000× TCID_50_ on postnatal day 15. After 45 days, mice were anesthetized using isoflurane. Blood was collected via cardiac puncture using a 22-g needle, allowed to clot at room temperature for 20 min, then centrifuged at 1,700 × *g* for 15 min. Serum was collected, pooled, then heated to 56°C for 30 min.

### Viral titer for EV-D68 in exosome-depleted medium.

The medium used for RD cells was 95% high-glucose Dulbecco’s modified eagle medium and 5% fetal bovine serum. The medium used for SH-SY5Y cells was 95% DMEM/nutrient mixture F-12 ham and 5% FBS. Standard FBS was used for control medium, and exosome-depleted FBS (System Biosciences [SBI], cat no. EXO-FBS-50A-1) was used for exosome-depleted medium. A549 Human non-small cell lung cancer exosomes from System Biosciences EXOP-120A were used to supplement exosome-depleted medium, and added at 1 μg/50 μL medium. Non-exosome associated MO47 virus at 1.20 and 1.24 g/cm^3^ was isolated from cell supernatant and mixed with either control or exosome-depleted medium before being added to TCID_50_ plates containing either RD or SH-SY5Y cells. These were incubated at 33°C.

### MTT assay.

SH-SY5Y neuroblastoma cells (purchased from ATCC CRL-2266) were grown in a 6-well tissue culture plate. Four wells were infected with 100 MOI of EV-D68 strain US/MO/14-18947, with a fifth well as an uninfected control. At days 3, 6, 9, or 12, the appropriate wells were rinsed with PBS, and medium plus 0.5 mg/mL MTT [3-(4,5-dimethylthiazol-2-yl)-2,5-diphenyltetrazolium bromide] was added for 60 min at 37°C. Images were taken after 1 h and notes were taken on the degree of cytopathic effect.

### Protocol for serial TCID_50_ measurements in SH-SY5Y cells.

SH-SY5Y neuroblastoma cells (purchased from ATCC CRL-2266) were plated at 3,000 cells/mL and grown in T182 tissue culture flasks (adherent and floating cells were observed). After 72 h, cells were infected with 100 MOI of EV-D68 strain US/MO/14-18947 at 33°C. After 1 h of incubation with virus, supernatant was removed (along with floating cells), adherent cells rinsed with PBS, and 40 mL of media (95% high-glucose DMEM + 5% fetal bovine serum) was added onto cells for 12 days at 33°C. At 3-day intervals, 10 mL of medium was removed from flask and frozen prior to density centrifugation and analysis.
